# Effect of endometrial thickness and embryo quality on live-birth rate of fresh IVF/ICSI cycles: a retrospective cohort study

**DOI:** 10.1186/s12958-020-00636-6

**Published:** 2020-08-21

**Authors:** Hong Lv, Xiuzhu Li, Jiangbo Du, Xiufeng Ling, Feiyang Diao, Qun Lu, Shiyao Tao, Lei Huang, Shiyao Chen, Xiumei Han, Kun Zhou, Bo Xu, Xiaoyu Liu, Hongxia Ma, Yankai Xia, Hongbing Shen, Zhibin Hu, Guangfu Jin, Yichun Guan, Xinru Wang

**Affiliations:** 1 State Key Laboratory of Reproductive Medicine, Nanjing Medical University, Nanjing, 211166 China; 2 Department of Epidemiology, School of Public Health, Nanjing Medical University, Nanjing, 211166 China; 3 Department of Reproduction, the Affiliated Nanjing Maternity and Child Health Hospital of Nanjing Medical University, Nanjing, 210004 China; 4 Department of Reproduction, the First Affiliated Hospital with Nanjing Medical University, Nanjing, 210029 China; 5 Department of Reproduction, Henan Medical Maternity and Child Health Care Hospital, Henan, 450052 China

**Keywords:** Endometrial thickness, Embryo quality, IVF/ICSI, Live-birth rate per cycle, Interaction

## Abstract

**Background:**

Successful implantation and delivery require both the functional embryo and receptive endometrium in assisted reproductive technology (ART) cycles. However, little is known about embryo-endometrial interaction on live-birth. We aimed to investigate the independent effect and interaction of endometrial thickness (EMT) and embryo quality on live-birth in fresh embryo transfer (ET) cycles.

**Methods:**

We conducted a retrospective cohort study including 15,012 ART cycles between 2013 and 2016 in three centers in China. Poisson regression with generalized estimating equations was employed to calculate relative risks (RRs) and 95% confidence intervals (CIs). We estimated the interaction of embryo quality and EMT on live-birth rate (LBR).

**Results:**

The LBR per cycle was 42.8% overall. LBR increased with increasing EMT and reached a plateau (50.6 to 54.2%) when EMT was 11 mm or thicker. Embryo quality represented by cumulative score was associated with LBR independently of number of embryos transferred and EMT. LBR was not increased with thicker EMT when only Q1 cleavage-stage embryo transferred (aRR 0.95, 95%CI 0.61–1.46). LBR was not increased significantly with thicker EMT with transfer of two good-quality cleavage-stage embryos and any blastocyst combination except Q1 group. There was significant interaction between EMT and embryo quality on LBR for cleavage-stage ETs (*P*=0.023).

**Conclusions:**

This study demonstrated the nonlinear EMT-LBR association and the EMT cut-off value of 11 mm which may be of more clinical significance for predicting live-birth. Embryo quality is an independent prognostic tool for LBR. Our finding of significant embryo-endometrial interaction indicates combination of EMT and embryos quality might improve the prognostic value in clinical practice for live-birth in patients undergoing transfer of 1–2 fresh cleavage-stage embryos.

## Background

Over the past decades, advances in clinical and laboratory techniques have substantially improved pregnancy rates and live-birth rates for assisted reproductive technology (ART). However, as reported by the Centers for Disease Control in the United States by the end of 2012, only 38.1% of all transfer cycles could result in live births [1]. Maternal age has been proved as the most predominant predictor for successful ART outcome [2]. During the process of ART treatment, the endometrial receptivity, and the number and quality of embryos transferred are also regarded as the key determinants for the outcome of in vitro fertilization (IVF)/intracytoplasmic sperm injection (ICSI) cycles [3–5].

As an indicator of endometrial receptivity, endometrial thickness (EMT) on the day of human chorionic gonadotropin (hCG) administration has been reported and reaffirmed as a potential prognostic tool for ART outcomes in multiple studies [6–8], despite significant advancements of ultrasonic [9], immunologic [10] and molecular [11] markers for endometrial receptivity. Although it remains a controversial issue, it has been widely suggested that a thin endometrium is associated with lower chance to conceive after IVF/ICSI, with cut-off values of EMT varying 7–9 mm in earlier studies [6, 12]. Also, several investigators have addressed the question whether there is a threshold value for thickness above which implantation is unlikely to occur, but the conclusion is contradictory [13, 14]. In a one-center study of 3350 IVF cycles, the non-linear association of EMT with live-birth was shown and required verification [7].

The number and quality of embryos transferred are the most important factors for IVF/ICSI outcome [15, 16]. Embryo morphological features assessed by optical microscopy are routine criteria applicable for embryo quality evaluation [17]. Several classification systems for embryo quality have been developed, and embryos have been graded into three categories: Good-quality, Fair-quality and Poor-quality [18, 19]. Evidences support the positive role of good-quality embryo for a better outcome of IVF [20, 21]. In addition, although two embryos transferred in one cycle have higher probability of live-birth than one embryo, multiple pregnancy may lead to the elevation of maternal and neonatal risks [22]. Therefore, to determine the optimal number and quality of embryos to transfer, cumulative embryo score (CES) and other embryo scoring methods based on the number of good-quality embryos transferred have been proposed [15, 23, 24] to predict pregnancy outcomes. However, the efficacy of these embryo scoring systems needs more optimization, because the association between quality of different embryo combinations and live-birth was not taken into consideration.

Successful pregnancy depends upon implantation, a complex process involving reciprocal interactions between the receptive endometrium and functional embryo in ART cycles. However, little is known about this embryo-endometrial interaction on live-birth. Thus, we hypothesized that the combination of EMT and embryo quality may improve the prognostic value in clinical practice for live-birth. Therefore, this multicenter study was designed to assess the association of EMT, embryo quality with live-birth independently and their interaction on live-birth based on a large scale of IVF/ICSI cycles.

## Methods

### Study population

**Fig. 1:**
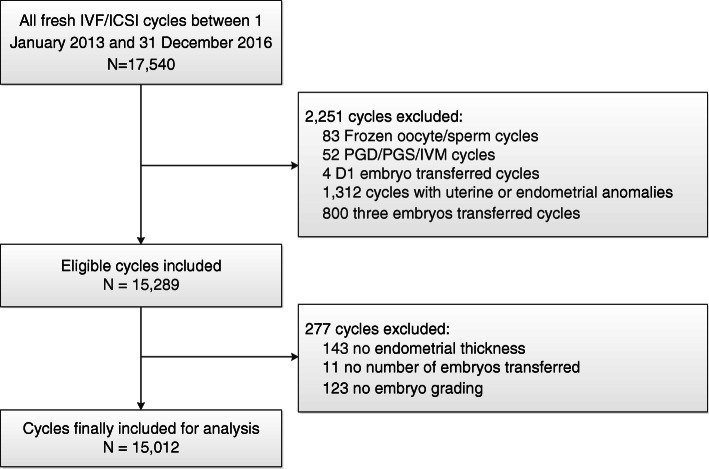
Flowchart of included and excluded cycles

A retrospective cohort study was conducted in three reproductive centers in China, including the First Affiliated Hospital with Nanjing Medical University (the First Affiliated Hospital of NMU), the Affiliated Nanjing Maternity and Child Health Hospital of Nanjing Medical University (NMU Affiliated Maternity Hospital) and Henan Medical Maternity and Child Health Care Hospital. All fresh IVF/ICSI embryo transfer (ET) cycles (*N*=17,540) between January 2013 and December 2016 were included. Exclusion criteria were as follows: 1) frozen oocyte or sperm, 2) preimplantation genetic diagnosis (PGD) or preimplantation genetic screening (PGS) or in vitro maturation (IVM), 3) D1 embryo transferred, 4) uterine or endometrial anomalies, 5) three or more embryos transferred, 6) cycles with missing data of EMT, number of embryos transferred and embryo grading. Finally, a total of 15,012 ET cycles were included for analysis. The flowchart is described in detail in Fig. 1.

### Baseline characteristics

Baseline characteristics of couples including demographics and fertility characteristics were obtained from the electronic medical records of reproductive centers. Demographics included maternal age and body mass index (BMI). Fertility characteristics included basal serum levels of follicle stimulating hormone (FSH), luteinizing hormone (LH), testosterone (T) and prolactin (PRL), duration of infertility, infertility type and causes of infertility. Primary infertility was defined as the inability to achieve a clinical pregnancy after 12 months of unprotected and regular sexual intercourse when a woman has never conceived, while secondary infertility was the incapability to conceive in a couple who have had at least one successful clinical pregnancy previously. The cause of infertility was categorized as male factor, tubal factor, anovulation factor and unexplained factor.

### Treatment characteristics

The parameters related to treatment included gonadotropin (Gn) dosage and duration, cycle protocols, fertilization methods (IVF, ICSI, or mixed IVF ICSI), serum level of estradiol (E2) and progesterone (P) on hCG day, year of transfer (2013, 2014, 2015 or 2016), stage of embryos transferred (cleavage-stage embryo or blastocyst), number of embryos transferred (1 or 2) and oocytes retrieved. Controlled ovarian hyperstimulation (COH) protocols were divided into six categories, including long agonist protocol, short agonist protocol, antagonist protocol, minimal-stimulation protocol, ultra-long protocol and other protocol [25].

### Endometrial thickness and embryo quality

EMT was measured in the midsagittal plane of the uterine body on the day of HCG administration. To facilitate the application of the results into clinical practice, we assigned EMT as regular 2-mm-intervalled categories (<7 mm, ≥7 to <9 mm, ≥9 to <11 mm, ≥11 to <13 mm, ≥13 to <15 mm, ≥15 to <17 mm, ≥17 mm). Cleavage-stage embryos were graded in three categories (Good, Fair and Poor) according to ASEBIR embryo assessment criteria [18], taking into account seven parameters (Day, Cell number, Fragmentation, Symmetry, Multi-nucleation, Vacuoles, Zona pellucida). In a combination of the stage of embryo development and the morphologic grade of the inner cell mass and trophectoderm, blastocysts were divided into three groups based on consensus scoring system for blastocysts: Good (1-6AA, 3–6AB, 3–6BA), Fair (3–6BB, 3–6 AC, 3–6CA, 1–2AB, 1–2BA), and Poor (1–6 BC, 1–6CB, 1–6CC, 1–2BB) [21]. With reference to cumulative embryo score (CES), we derived cumulative score by adding the individual quality score of all the embryos transferred (Poor =1 point, Fair =2 points, Good =3 points) to determine the current group of cleavage-stage embryo quality (Q1 =1 point, Q2 =2 points, Q3 =3 points, Q4 =4 points, Q5 =5 points, Q6 =6 points). In consideration of the limited blastocyst cycles, we finalized the current group of blastocyst embryo quality (Q1 =1 point, Q2 =2 points, Q3 =3 points, Q4 =4/5/6 points). We have confirmed the efficiency of CES respectively for both cleavage-stage embryos and blastocysts by comparing the differences of LBR per cycle after transfers of different embryo combinations when ‘Good’ was set as the reference group in Additional file 1: Table S1.

### Outcomes

Live-birth was defined as the birth of at least one live born neonate beyond 24 weeks of gestational age. The live-birth rate (LBR) per cycle was defined as the rate of achieving a live birth from a fresh ET cycle.

### Statistical analysis

Differences between demographics and clinical characteristics of patients undergoing ART treatment were calculated by t-test or Kruskal-Wallis test as appropriate for continuous variables and chi-square test for categorical variables. Poisson regression with generalized estimating equations was employed to calculate relative risks (RRs) and 95% confidence intervals (CIs) to estimate the association of live-birth after IVF/ICSI treatment with embryo score and EMT. To adjust for the possible dependence in outcome introduced by repeated cycles in the same couple, we constructed models with couple’s identification number as a cluster. For the association between EMT and live-birth, we selected group EMT of ≥11 to <13 mm as the reference. We adjusted the multiple models for potential confounders including maternal age (continuous variable), maternal BMI (continuous variable), basal FSH (continuous variable), basal LH (continuous variable), basal T (continuous variable), basal PRL (continuous variable), duration of infertility (continuous variable), infertility type (primary/secondary), male factor of infertility (yes/no), anovulation factor of infertility (yes/no), Gn dosage (continuous variable), Gn duration (continuous variable), cycle protocols (long agonist protocol/short agonist protocol/antagonist protocol/minimal-stimulation protocol/ultra-long protocol/other protocol), E2 on hCG day (continuous variable), year of transfer (2013/2014/2015/2016), oocytes retrieved (continuous variable), number of embryos transferred (1/2), embryo quality as well as location of fertility centers to control heterogeneity of multicenter. To test whether the association of embryo quality with live-birth was independent of number of embryos transferred, we ran separate models with and without number of embryos transferred as a sensitivity analysis. Specific subgroup analyses on embryo stage were performed to estimate its potential influence on these relationships. Furthermore, we estimated the multiplicative interaction of EMT and embryo quality on LBR per cycle. Given the heterogeneity in the stimulation protocols for COH, sensitivity analysis was also performed by restricting the analysis to long agonist protocol, the most common type of protocol. Data analysis was conducted using R software (Version 3.3.3, 2017-03-06; R Foundation for Statistical Computing, http://www.cran.r-project.org/). Statistical significance was interpreted as *P*-value <0.05.

## Results

In total, 15,012 IVF/ICSI cycles from 13,909 patients were analyzed during the study period. Table 1 summarized basic characteristics of the patients undergoing ART treatment who were 31.23 years old on average. The LBR per cycle was 42.8% overall. LBR per cycle was higher in patients with primary infertility compared with secondary type (45.4% vs. 39.5%). Patients receiving ultra-long protocol showed highest LBR per cycle (52.3%) in all COH protocols. LBR per cycle elevated from 39.5 to 46.2% with year of transfer. 94.8% of cycles transferred cleavage-stage embryos while only 5.2% transferred blastocysts. 71.7% of cycles had two embryos transferred while 28.3% had a single ET, and after comparisons, LBR per cycle was significantly higher for two ETs than only one embryo (48.2% vs. 29.1%, *P* <0.001).

**Table 1:** Basic characteristics of the patients undergoing IVF/ICSI treatment

Characteristics	All cycles (***n***=15,012)	LBR per cycle (%)
**Demographics**		
Maternal age, years, mean (SD)^*^	31.23 (5.29)	
Maternal BMI, kg/m^2^, mean (SD)^*^	22.65 (3.26)	
**Fertility characteristics**		
Basal FSH, IU/L, mean (SD)^*^	7.46 (6.23)	
Basal LH, IU/L, mean (SD)^*^	4.58 (6.33)	
Basal T, ng/ml, mean (SD)^*^	10.21 (16.44)	
Basal PRL, ng/ml, mean (SD)^*^	23.98 (222.33)	
Duration of infertility, years, mean (SD)^*^	3.97 (3.20)	
Infertility type, n (%)^*^		
Primary	8314 (55.4)	45.4
Secondary	6695 (44.6)	39.5
Male factor of infertility, n (%)^*^	6193 (41.3)	44.9
Tubal factor of infertility, n (%)	8799 (58.6)	42.2
Anovulation factor of infertility, n (%)^*^	1714 (11.4)	47.1
Unexplained infertility, n (%)	746 (5.0)	46.1
**Treatment characteristics**		
Gn dosage, IU, mean (SD)^*^	1920.99 (1007.41)	
Gn duration, days, mean (SD)^*^	10.14 (3.35)	
Cycle protocols, n (%)^*^		
Long agonist protocol	9579 (64.4)	49.4
Short agonist protocol	1176 (7.9)	28.9
Antagonist protocol	1581 (10.6)	39.7
Minimal-stimulation protocol	940 (6.3)	19.7
Ultra-long protocol	434 (2.9)	52.3
Other protocol	1153 (7.8)	23.9
Fertilization methods, n (%)		
IVF	10,401 (69.3)	42.6
ICSI	4074 (27.2)	43.7
Mixed IVF ICSI	525 (3.5)	41.1
E2 on hCG day, pg/ml, mean (SD)^*^	3289.68 (1915.22)	
P on hCG day, ng/ml, mean (SD)	1.21 (18.29)	
Year of transfer, n (%)^*^		
2013	3781 (25.2)	39.5
2014	3894 (25.9)	41.7
2015	3862 (25.7)	44.1
2016	3475 (23.1)	46.2
Stage of embryos transferred, n (%)		
Cleavage-stage embryo (D2/D3/D4)	14,236 (94.8)	42.8
Blastocyst (D5/D6/D7)	776 (5.2)	43.9
Number of embryos transferred, n (%)^*^		
1	4254 (28.3)	29.1
2	10,758 (71.7)	48.2
Oocytes retrieved, mean (SD)^*^	8.17 (4.91)	

Abbreviations: *LBR* Live-birth rate, *BMI* Body mass index, *SD* Standard deviation

^*^ *P*<0.05 for comparisons between live-birth group and no live birth group, chi-square test was used for categorical variables, and t-test or Kruskal–Wallis test for continuous variables

### Effect of EMT on LBR of fresh IVF/ICSI cycles

**Fig. 2:**
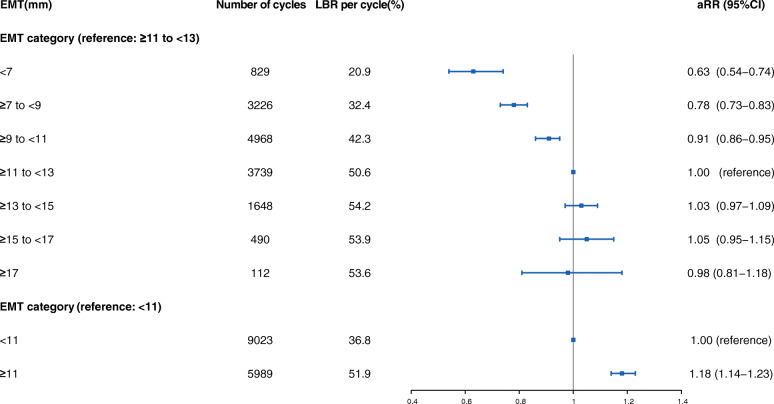
Adjusted relative risk for live-birth according to EMT categories. Analysis Adjusted for maternal age, maternal BMI, basal FSH, basal LH, basal T, basal PRL, duration of infertility, infertility type, male factor of infertility, anovulation factor of infertility, Gn dosage, Gn duration, cycle protocols, E2 on hCG day, year of transfer, oocytes retrieved, location of fertility centers, number of embryos transferred and embryo score. Abbreviations: LBR, live-birth rate; aRR, adjusted relative risk; CI, confidence interval

The LBR increased with increasing EMT on hCG day as either a continuous variable or ordinal categories after adjusting the confounders (both *P*_trend_ <0.001, data not shown). As shown in Fig. 2**,** compared with EMT ≥11 to <13 mm, EMT less than 7 mm, ≥7 to <9 mm, ≥9 to <11 mm were associated with decreased LBR of 37, 22 and 9%, respectively. LBR per cycle reached a plateau (50.6 to 54.2%), when EMT was 11 mm or thicker. No differences were noted when EMT was ≥13 to <15 mm (aRR 1.03, 95%CI 0.97–1.09), ≥15 to <17 mm (1.05, 0.95–1.15) and more than 17 mm (0.98, 0.81–1.18) in the adjusted model. Hence, the cut-off value for EMT was set as 11 mm. We observed that women with EMT ≥11 mm enjoyed an increased LBR compared with EMT <11 mm, with aRR of 1.18 (95%CI 1.14–1.23).

### Effect of embryo quality on LBR of fresh IVF/ICSI cycles

In Table 2, we observed that LBR significantly increased as cleavage-stage embryo quality elevated (*P*_for trend_ <0.001). In sensitivity analysis, similar results were observed when the analysis was adjusted for the number of embryos transferred. Additional file 1: Table S2 also showed LBR significantly increased as blastocyst quality elevated (*P*_for trend_ <0.001) with and without number of embryos transferred. The association of embryo quality with live-birth remained unchanged after adjustment for EMT regardless of cleavage-stage embryo or blastocyst transfer.

**Table 2:** Adjusted relative risk for live-birth according to cleavage-stage embryo quality

Embryo quality	Number of cycles	Number of live-birth (LBR per cycle, %)	aRR (95%CI) ^**a**^	aRR (95%CI) ^**b**^	aRR (95%CI) ^**c**^
Q1 (1 point)	770	104 (13.5)	1.00 (reference)	1.00 (reference)	1.00 (reference)
Q2 (2 points)	2620	802 (30.6)	**1.99 (1.62–2.44)**	**1.94 (1.57–2.39)**	**1.93 (1.56–2.38)**
Q3 (3 points)	3315	1348 (40.7)	**2.43 (1.98–2.98)**	**2.35 (1.91–2.90)**	**2.34 (1.89–2.88)**
Q4 (4 points)	3154	1590 (50.4)	**2.88 (2.35–3.53)**	**2.74 (2.21–3.40)**	**2.72 (2.19–3.38)**
Q5 (5 points)	2993	1629 (54.4)	**3.07 (2.50–3.76)**	**2.92 (2.35–3.62)**	**2.90 (2.33–3.60)**
Q6 (6 points)	1384	613 (44.3)	**3.27 (2.64–4.06)**	**3.12 (2.48–3.91)**	**3.10 (2.47–3.89)**
*P*_for trend_			**<0.001**	**<0.001**	**<0.001**

Abbreviations: *LBR* Live-birth rate, *aRR* Adjusted relative risk, *CI* Confidence interval

^a^ Adjusted for maternal age, maternal BMI, Basal FSH, Basal LH, Basal T, Basal PRL, duration of infertility, infertility type, male factor of infertility, anovulation factor of infertility, Gn dosage, Gn duration, cycle protocols, E2 on hCG day, year of transfer, oocytes retrieved and location of fertility centers

^b^ Adjusted for maternal age, maternal BMI, basal FSH, basal LH, basal T, basal PRL, duration of infertility, infertility type, male factor of infertility, anovulation factor of infertility, Gn dosage, Gn duration, cycle protocols, E2 on hCG day, year of transfer, oocytes retrieved, location of fertility centers and number of embryos transferred

^c^ Adjusted for maternal age, maternal BMI, basal FSH, basal LH, basal T, basal PRL, duration of infertility, infertility type, male factor of infertility, anovulation factor of infertility, Gn dosage, Gn duration, cycle protocols, E2 on hCG day, year of transfer, oocytes retrieved, location of fertility centers, number of embryos transferred and EMT

### Interaction of EMT and embryo quality on live-birth

We also analyzed the relationship between cleavage-stage embryo quality and live-birth stratified by EMT and the relationship between EMT and live-birth stratified by cleavage-stage embryo quality (Table 3). We found LBR increased in higher embryo quality group than Q1 (poor) group in patients with EMT <11 mm or EMT ≥11 mm. Stratified by cleavage-stage embryo quality, we found LBR did not increase with thicker EMT when only Q1 embryo (aRR 0.95, 95%CI 0.61–1.46) or Q6 embryos (aRR 1.12, 95%CI 0.98–1.27) were for transfer, but LBR did increase with thicker EMT in other groups of embryo quality. Test for multiplicative interaction was significant (*P*_for interaction_ =0.023). While for blastocyst transfers, we found LBR in Q3 and Q4 embryo quality group significantly increased compared with Q1 group when EMT was ≥11 mm in Additional file 1: Table S3. When stratified by embryo quality, LBR did not increase significantly with thicker EMT in groups of embryo quality except Q1 group. Statistical interaction between EMT and blastocyst embryo quality on live-birth was not significant (*P*=0.860). The sensitivity analysis restricted to long agonist protocol was shown in Additional file 1: Table S4. No remarkable changes in results were found in the stratification analysis for cleavage-stage ET cycles although test for multiplicative interaction was not significant (*P*=0.195).

**Table 3:** Adjusted relative risk for live-birth according to cleavage-stage embryo quality stratified by EMT, and EMT stratified by cleavage-stage embryo quality

EMT	Q1 (***n***=770)	Q2 (***n***=2620)	Q3 (***n***=3315)	Q4 (***n***=3154)	Q5 (***n***=2993)	Q6 (***n***=1384)
**<11 mm**						
N live-births/N cycles (LBR per cycle, %)	68/574 (11.8)	457/1775 (25.7)	759/2104 (36.1)	754/1702 (44.3)	803/1642 (48.9)	318/796 (39.9)
aRR (95%CI) ^a^	1.00 (reference)	**1.86 (1.43–2.41)**	**2.38 (1.84–3.09)**	**2.85 (2.17–3.75)**	**3.13 (2.38–4.12)**	**3.67 (2.74–4.91)**
**≥11 mm**						
N live-births/N cycles (LBR per cycle, %)	36/196 (18.4)	345/845 (40.8)	589/1211 (48.6)	836/1452 (57.6)	826/1351 (61.1)	295/588 (50.2)
aRR (95%CI) ^a^	1.00 (reference)	**2.05 (1.43–2.94)**	**2.35 (1.64–3.37)**	**2.68 (1.86–3.86)**	**2.78 (1.93–4.00)**	**2.75 (1.89–4.01)**
aRR (95%CI) ^b^	0.95 (0.61–1.46)	**1.23 (1.09–1.40)**	**1.18 (1.07–1.29)**	**1.22 (1.13–1.32)**	**1.17 (1.08–1.26)**	1.12 (0.98–1.27)

Abbreviations: *N* Number, *LBR* Live-birth rate, *aRR* Adjusted relative risk, *CI* Confidence interval

^a^ Adjusted for maternal age, maternal BMI, basal FSH, basal LH, basal T, basal PRL, duration of infertility, infertility type, male factor of infertility, anovulation factor of infertility, Gn dosage, Gn duration, cycle protocols, E2 on hCG day, year of transfer, oocytes retrieved, location of fertility centers and number of embryos transferred. Reference was Q1 group

^b^ Adjusted for maternal age, maternal BMI, basal FSH, basal LH, basal T, basal PRL, duration of infertility, infertility type, male factor of infertility, anovulation factor of infertility, Gn dosage, Gn duration, cycle protocols, E2 on hCG day, year of transfer, oocytes retrieved, location of fertility centers and number of embryos transferred. Reference was EMT <11 mm group

## Discussion

In the current study, we confirmed the nonlinearity in EMT-LBR association and a plateau of LBR per cycle when EMT was 11 mm or thicker. The embryo quality graded by cumulative score was significantly associated with LBR for cleavage-stage and for blastocyst ETs, and these associations were independent of the number of embryo transferred and EMT. To the best of our knowledge, this is the first multicenter retrospective cohort study to evaluate the independent and interaction effects of EMT on hCG day and embryo quality on the outcome of fresh IVF/ICSI cycles.

Our results are consistent with the reported association of thin EMT with poor pregnancy outcomes in fresh ET cycles [4, 7, 12], while a consensus is still lacking on what the precise definition of thin endometrium. The present study showed EMT above 11 mm is related to a higher chance of delivery. This finding indicates that adequate endometrial development is favorable for improving chance of live-birth, which concurs to some extent with one recent study [12]. As discussed in the meta-analysis, a thickness threshold of 7 mm was frequently reported below which pregnancy rates decreased rapidly, but the case of EMT below 7 mm occurred infrequently [6] and the threshold was not available for live-birth evaluation. Therefore, there may be of more clinical significance for the EMT cut-off value of 11 mm, which could be more helpful for decision making on fresh ET or freezing of embryos for potentially better chances to conceive with an endometrium developed under natural conditions in subsequent cryo-cycles. However, some others could not establish a significant correlation between EMT and the chance to conceive in the study population with euploid ETs [26]. Debate on the predictive value EMT in clinical outcomes is ongoing [6]. In order to investigate the real endometrium’s effect, it is interesting to investigate the value of EMT in patients undergoing frozen embryo transfer cycles in the further study because in this population the possible negative effect of ovarian hyperstimulation on endometrial development is absent.

The mechanism of the association between thin endometrium and difficulty for implantation and development remains elusive. One speculation relates to that the implanting embryo would be much closer to the basal layer endometrium with higher oxygen concentrations in patients with thin functional layer EMT [27]. It is well known that high oxygen tensions could be detrimental for embryo implantation and development due to the production of reactive oxygen species [28]. In addition, it has been speculated that low late-follicular estradiol levels could result in inadequate endometrial proliferation [7, 29]. Follicular estrogen production is a reflection of follicular maturity and impacts both endometrial receptivity and follicular growth, oocyte maturation, sperm transport, embryo survival [30]. In other words, it is possible that the observed reduction in pregnancy rates associated with a thin endometrium may be a consequence of another unmeasured aspect of oocyte/embryo quality besides their morphologic grading. Furthermore, it is plausible that thin endometrium may be related to iatrogenic event like Asherman’s syndrome [31] or use of clomiphene citrate, leading to an E receptor abnormality [27] and the subsequent lack of a normal proliferative response to the rising E level.

The thickened endometrium provides a site for attachment, as well as the source of nourishment for an implanting embryo during its first few weeks, until the placenta starts to develop. Whether a thick endometrium has a detrimental effect on clinical outcome is controversial [12, 13]. Our findings indicated that thick EMT (>17 mm) neither played an adverse role nor conferred additional benefit, similar to results reported in recent studies [12, 32], which suggested a ceiling effect of EMT after excluding patients with intrauterine pathologies such as polyps or fibroids.

Owing to the adverse perinatal risks and multiple pregnancy related costs, increased use of elective single-embryo transfer (eSET) in couples attempting assisted conception has been proposed and gradually adopted by some countries, such as Sweden and Belgium [22]. In other countries, however, the adoption of eSET in practice is hampered due to the desire of both clinicians and patients to ensure pregnancy and to reduce the costs of multiple ART cycles, especially in countries where ART is not publicly funded [33]. Opinion should, therefore, shift towards the view that the decision to transfer one or two embryos should be made according to not only the number but quality of available embryos. With regard to single embryo quality, there was a robust correlation between morphologic parameters and live-birth in previous studies transferring one cleavage-stage embryo [34] or one blastocyst [35]. In general, our findings support the validity of morphologic grading to be used to guide the embryo selection.

In addition, we also have realized that the association of live birth with embryo quality of different embryo combinations is poorly understood, while it is imperative for decision-making of optimal embryos number and quality to transfer. CES and mean embryo score (MES) based on 4-point embryo score have been proposed to predict pregnancy outcomes restricted to cleavage-stage embryos transfer [17, 36]. Our data provided adequate support for the efficiency of CES (Poor =1 point, Fair =2 points, Good =3 points) in predicting live-birth when restricted to cleavage-stage ETs. For blastocyst transfer, it is also appropriate to determine the group of blastocyst embryo quality by cumulative score although we observed that a good-quality blastocyst transfer had similar LBR compared to that of a fair-quality blastocyst and combinations of blastocyst which was graded ‘Poor’ or ‘Fair’, which may be potentially caused by the limited cycles. To reduce the risk of multiple gestations, eSET was recommended to women with both one fair or good embryo and embryo pairs with the same cumulative score. Our findings doubted about other embryo scoring methods based on the number of good-quality embryos transferred [15] and grading of best embryo transferred [37] applied to distinguish different embryo combinations and to predict live-birth. Consequently, our findings filled the gaps of current embryo scoring systems and could help clinicians and infertile couples make a better choice.

It is estimated that embryos account for one-third of implantation failures, while suboptimal endometrial receptivity and altered embryo-endometrial dialogue are responsible for the remaining two-thirds [38]. A two-way communication between the embryo and receptive endometrium leads to implantation and the course of pregnancy, but ethical restrictions and the lack of mechanistic studies have delayed studies on embryo-endometrial interactions in humans [39]. Endometrial receptivity and selectivity are two complementary concepts introduced to describe the endometrium, the emerging concept as a biosensor of embryo quality [40]. Either impaired embryo development potential or impaired endometrial selectivity/receptivity has a negative effect on the embryo-endometrial cross-talk. It is gradually acknowledged that the thickness of the endometrium can be used as a noninvasive parameter to infer on endometrial receptivity. Therefore, the investigation of interaction between embryo quality and EMT would provide some evidence. Our research showed that LBR could not be improved by thicker EMT when only one poor cleavage-stage embryo is available for transfer. In this circumstance, it is recommendable to restart the treatment for better embryo quality. What’s more, the LBR did not increase significantly with thicker EMT with transfer of two good-quality cleavage-stage embryos and blastocysts in groups of embryo quality except in Q1 group. Our findings suggested that the requirement for optimal endometrial environment is less stringent when high quality embryos are available for transfer. It coincides with a previous study with a smaller sample size [29]. It seemed that the additional 2–3 days delay may provide an inadequate endometrium with additional time to develop. Taken together, the significant embryo-endometrial interaction on live-birth suggests that the combination of EMT and embryos quality may improve the prognostic value in clinical practice for live-birth in patients undergoing transfer of 1–2 fresh cleavage-stage embryos, and for blastocyst transfers, embryo quality may be more important for treatment outcome compared with EMT.

Our findings were strengthened by 2-mm EMT-LBR relationship evaluation in consideration of clinical value in practice [7], in addition to establish a cutoff value of 11 mm for endometrial thickness above which LBR per cycle reached a plateau. Relying on a large sample size, transfers of different combinations of embryos were first compared with a good-quality ET to predict live-birth in detail, which confirmed the efficiency of CES separately for both cleavage-stage embryos and blastocysts and might compensate for the deficiency of some embryo scoring methods and improve embryo selection. It is noteworthy that the independent effects were confirmed in a prediction model with analysis of 100 prospectively recorded variables [4], while this was the first time to estimate the interaction of endometrial characteristics and embryo quality on LBR of fresh IVF/ICSI cycles. Still, the weaknesses of this study included its retrospective nature of the data, the possibility of unidentified confounding variables which could not be excluded, and the reproducibility of EMT and embryo grade assessment of different observers in different centers. Moreover, due to only a few blastocyst transfer cycles were conducted before 2016 (only 5.2% included in this study), these findings of blastocyst transfer required verification in future studies based on a larger sample. The observations in our study were based on fresh cycles, so the conclusion may not apply to frozen cycles.

## Conclusions

In conclusion, this study demonstrated the nonlinearity in EMT-LBR association and the EMT cut-off value of 11 mm which might be of more clinical significance for predicting live-birth. Embryo quality represented by cumulative score was an independently prognostic tool for LBR. The significant embryo-endometrial interaction on live-birth suggested the combination of EMT and embryo quality might be useful in making prognostications about the potential for LBR in patients undergoing transfer of 1–2 fresh cleavage-stage embryos in view of a number of important and potential confounding variables. For blastocyst transfers, embryo quality might be more important for treatment outcome when compared with EMT.

## Supplementary information

**Additional file 1: Table S1.** Adjusted relative risk for live-birth according to embryo combinations. **Table S2.** Adjusted relative risk for live-birth according to blastocyst embryo quality. **Table S3.** Adjusted relative risk for live-birth according to blastocyst embryo quality stratified by EMT, and EMT stratified by blastocyst embryo quality. **Table S4.** Adjusted relative risk for live-birth according to embryo quality stratified by EMT, and EMT stratified by embryo quality in long agonist protocol.

## Data Availability

The datasets used and analyzed during the current study are available from the corresponding author on reasonable request.

## Abbreviations

ART: Assisted reproductive technology
EMT: Endometrial thickness
ET: Embryo transfer
RRs: Relative risks
CIs: Confidence intervals
LBR: Live-birth rate
IVF: In vitro fertilization
ICSI: Intracytoplasmic sperm injection
hCG: Human chorionic gonadotropin
CES: Cumulative embryo score
PGD: Preimplantation genetic diagnosis
PGS: Preimplantation genetic screening
IVM: In vitro maturation
BMI: Body mass index
FSH: Follicle stimulating hormone
LH: Luteinizing hormone
T: Testosterone
PRL: Prolactin
Gn: Gonadotropin
E2: Estradiol
P: Progesterone
COH: Controlled ovarian hyperstimulation
eSET: Elective single-embryo transfer
MES: Mean embryo score
