# Supplementary material for: Effect of endometrial thickness and embryo quality on live-birth rate of fresh IVF/ICSI cycles: a retrospective cohort study
Source: Reprod Biol Endocrinol. 2020 Aug 21;18:89. doi: 10.1186/s12958-020-00636-6 (PMC7441697; doi:10.1186/s12958-020-00636-6)
Supplement: Supplementary file 1 — Additional file 1: Table S1. Adjusted relative risk for live-birth according to embryo combinations. Table S2. Adjusted relative risk for live-birth according to blastocyst embryo quality. Table S3. Adjusted relative risk for live-birth according to blastocyst embryo quality stratified by EMT, and EMT stratified by blastocyst embryo quality. Table S4. Adjusted relative risk for live-birth according to embryo quality stratified by EMT, and EMT stratified by embryo quality in long agonist protocol. [file 12958_2020_636_MOESM1_ESM.docx]

Table S1. Adjusted relative risk for live-birth according to embryo combinations.

| **Embryo combinations**  **(score)** | **Cleavage-stage embryo (n=14236)** | | |  | **Blastocyst (n=776)** | | |
| --- | --- | --- | --- | --- | --- | --- | --- |
|  | **Number of cycles** | **Number of live-birth (LBR per cycle, %)** | **aRR (95% CI) ^a^** |  | **Number of cycles** | **Number of live-birth (LBR per cycle, %)** | **aRR (95% CI) ^a^** |
| Poor (1 point) | 770 | 104 (13.5) | **0.42 (0.34-0.51)** |  | 253 | 80 (31.6) | **0.67 (0.52-0.86)** |
| Fair (2 points) | 1493 | 383 (25.7) | **0.78 (0.69-0.88)** |  | 189 | 97 (51.3) | 0.89 (0.72-1.10) |
| Good (3 points) | 1393 | 494 (35.5) | 1.00 (reference) |  | 156 | 79 (50.6) | 1.00 (reference) |
| Poor+Poor (2 points) | 1127 | 419 (37.2) | **0.87 (0.78-0.98)** |  | 101 | 46 (45.5) | 0.99 (0.74-1.33) |
| Fair+Poor (3 points) | 1922 | 854 (44.4) | 1.02 (0.93-1.12) |  | 42 | 19 (45.2) | 0.97 (0.65-1.44) |
| Fair+Fair (4 points) | 2937 | 1483 (50.5) | **1.21 (1.11-1.32)** |  | 17 | 8 (47.1) | 1.02 (0.57-1.81) |
| Good+Poor (4 points) | 217 | 107 (49.3) | **1.19 (1.01-1.40)** |  | 1 | 1 (100.0) | **1.68 (1.08-2.62)** |
| Good+Fair (5 points) | 2993 | 1629 (54.4) | **1.28 (1.18-1.40)** |  | 10 | 6 (60.0) | 1.04 (0.55-1.96) |
| Good+Good (6 points) | 1384 | 613 (44.3) | **1.37 (1.22-1.54)** |  | 7 | 5 (71.4) | **2.06 (1.40-3.05)** |

Abbreviations: LBR, live-birth rate; aRR, adjusted relative risk; CI, confidence interval.

^a^ Adjusted for maternal age, maternal BMI, Basal FSH, Basal LH, Basal T, Basal PRL, duration of infertility, infertility type, male factor of infertility, anovulation factor of infertility, Gn dosage, Gn duration, cycle protocols, E2 on hCG day, year of transfer, oocytes retrieved and location of fertility centers.

Table S2. Adjusted relative risk for live-birth according to blastocyst embryo quality.

| **Embryo quality** | **Number of cycles** | **Number of live-birth (LBR per cycle, %)** | **aRR (95% CI) ^a^** | **aRR (95% CI) ^b^** | **aRR (95% CI) ^c^** |
| --- | --- | --- | --- | --- | --- |
| Q1 (1 points) | 253 | 80 (31.6) | 1.00 (reference) | 1.00 (reference) | 1.00 (reference) |
| Q2 (2 points) | 290 | 143 (49.3) | **1.39 (1.10-1.74)** | **1.36 (1.12-1.72)** | **1.38 (1.09-1.74)** |
| Q3 (3 points) | 198 | 98 (49.5) | **1.49 (1.17-1.91)** | **1.48 (1.15-1.90)** | **1.49 (1.16-1.90)** |
| Q4 (4/5/6 points) | 35 | 20 (57.1) | **1.81 (1.24-2.65)** | **1.70 (1.10-2.64)** | **1.78 (1.16-2.73)** |
| *P* for trend |  |  | **<0.001** | **0.002** | **0.004** |

Abbreviations: LBR, live-birth rate; aRR, adjusted relative risk; CI, confidence interval.

^a^ Adjusted for maternal age, maternal BMI, Basal FSH, Basal LH, Basal T, Basal PRL, duration of infertility, infertility type, male factor of infertility, anovulation factor of infertility, Gn dosage, Gn duration, cycle protocols, E2 on hCG day, year of transfer, oocytes retrieved and location of fertility centers.

^b^ Adjusted for maternal age, maternal BMI, basal FSH, basal LH, basal T, basal PRL, duration of infertility, infertility type, male factor of infertility, anovulation factor of infertility, Gn dosage, Gn duration, cycle protocols, E2 on hCG day, year of transfer, oocytes retrieved , location of fertility centers and number of embryos transferred.

^c^ Adjusted for maternal age, maternal BMI, basal FSH, basal LH, basal T, basal PRL, duration of infertility, infertility type, male factor of infertility, anovulation factor of infertility, Gn dosage, Gn duration, cycle protocols, E2 on hCG day, year of transfer, oocytes retrieved , location of fertility centers, number of embryos transferred and EMT.

Table S3. Adjusted relative risk for live-birth according to blastocyst embryo quality stratified by EMT, and EMT stratified by blastocyst embryo quality.

| **EMT** | **Q1 (n=253)** | **Q2 (n=290)** | **Q3 (n=198)** | **Q4 (n=35)** |
| --- | --- | --- | --- | --- |
| **<11 mm** |  |  |  |  |
| N live-births/N cycles (LBR per cycle, %) | 37/154 (24.0) | 66/147 (44.9) | 46/110 (41.8) | 9/19 (47.4) |
| aRR (95%CI) ^a^ | 1.00 (reference) | 1.48 (0.99-2.22) | 1.52 (0.99-2.33) | 1.41 (0.70-2.86) |
| **≥11 mm** |  |  |  |  |
| N live-births/N cycles (LBR per cycle, %) | 43/99 (43.4) | 77/143 (53.8) | 52/88 (59.1) | 11/16 (68.8) |
| aRR (95%CI) ^a^ | 1.00 (reference) | 1.33 (0.99-1.78) | **1.49 (1.10-2.01)** | **2.24 (1.35-3.73)** |
| aRR (95%CI) ^b^ | **1.54 (1.00-2.35)** | 1.13 (0.88-1.46) | 1.30 (0.96-1.76) | 1.00 (0.27-3.65) |

Abbreviations: N, number; LBR, live-birth rate; aRR, adjusted relative risk; CI, confidence interval.

^a^ Adjusted for maternal age, maternal BMI, basal FSH, basal LH, basal T, basal PRL, duration of infertility, infertility type, male factor of infertility, anovulation factor of infertility, Gn dosage, Gn duration, cycle protocols, E2 on hCG day, year of transfer, oocytes retrieved, location of fertility centers and number of embryos transferred. Reference was Q1 group.

^b^ Adjusted for maternal age, maternal BMI, basal FSH, basal LH, basal T, basal PRL, duration of infertility, infertility type, male factor of infertility, anovulation factor of infertility, Gn dosage, Gn duration, cycle protocols, E2 on hCG day, year of transfer, oocytes retrieved, location of fertility centers and number of embryos transferred. Reference was EMT<11mm group.

Table S4. Adjusted relative risk for live-birth according to embryo quality stratified by EMT, and EMT stratified by embryo quality in long agonist protocol.

| **EMT** | **Cleavage-stage embryo quality (n=8997)** | | | | | |  | **Blastocyst quality (n=582)** | | | |
| --- | --- | --- | --- | --- | --- | --- | --- | --- | --- | --- | --- |
|  | **Q1 (n=299)** | **Q2 (n=1330)** | **Q3 (n=1896)** | **Q4 (n=2373)** | **Q5 (n=2228)** | **Q6 (n=871)** |  | **Q1 (n=160)** | **Q2 (n=242)** | **Q3 (n=153)** | **Q4 (n=27)** |
| **<11 mm** |  |  |  |  |  |  |  |  |  |  |  |
| N live-births/N cycles (LBR per cycle, %) | 31/171 (18.1) | 242/692 (35.0) | 431/981 (43.9) | 530/1157 (45.8) | 577/1130 (51.1) | 208/462 (45.0) |  | 19/73  (26.0) | 54/118 (45.8) | 37/77  (48.1) | 6/12  (50.0) |
| aRR (95%CI) ^a^ | 1.00 (reference) | **1.74 (1.19-2.52)** | **2.12 (1.46-3.07)** | **2.38 (1.63-3.49)** | **2.64 (1.80-3.87)** | **2.89 (1.95-4.30)** |  | 1.00 (reference) | **1.86 (1.13-3.07)** | **1.95 (1.16-3.26)** | 1.63 (0.69-3.84) |
| **≥11 mm** |  |  |  |  |  |  |  |  |  |  |  |
| N live-births/N cycles (LBR per cycle, %) | 27/128 (21.1) | 288/638 (45.1) | 483/915 (52.8) | 726/1216 (59.7) | 685/1098 (62.4) | 219/409 (53.5) |  | 41/87  (47.1) | 70/124 (56.5) | 47/76  (61.8) | 11/15  (73.3) |
| aRR (95%CI) ^a^ | 1.00 (reference) | **1.95 (1.30-2.91)** | **2.25 (1.51-3.35)** | **2.57 (1.71-3.86)** | **2.60 (1.73-3.91)** | **2.61 (1.72-3.91)** |  | 1.00 (reference) | 1.34 (1.00-1.80) | **1.48 (1.10-2.00)** | **2.30 (1.38-3.86)** |
| aRR (95%CI) ^b^ | 1.29 (0.77-2.16) | **1.23 (1.06-1.41)** | **1.18 (1.07-1.29)** | **1.25 (1.15-1.36)** | **1.16 (1.07-1.26)** | 1.07 (0.93-1.23) |  | 1.80 (0.92-3.51) | 1.16 (0.89-1.51) | 1.32 (0.97-1.81) | 0.02 (0.00-893.70) |

Abbreviations: N, number; LBR, live-birth rate; aRR, adjusted relative risk; CI, confidence interval.

^a^ Adjusted for maternal age, maternal BMI, basal FSH, basal LH, basal T, basal PRL, duration of infertility, infertility type, male factor of infertility, anovulation factor of infertility, Gn dosage, Gn duration, E2 on hCG day, year of transfer, oocytes retrieved, location of fertility centers and number of embryos transferred. Reference was Q1 group.

^b^ Adjusted for maternal age, maternal BMI, basal FSH, basal LH, basal T, basal PRL, duration of infertility, infertility type, male factor of infertility, anovulation factor of infertility, Gn dosage, Gn duration, E2 on hCG day, year of transfer, oocytes retrieved, location of fertility centers and number of embryos transferred. Reference was EMT<11mm group.
